# Epigallocatechin gallate (EGCG) suppresses growth and tumorigenicity in breast cancer cells by downregulation of miR-25

**DOI:** 10.1080/21655979.2019.1657327

**Published:** 2019-08-29

**Authors:** Lingling Zan, Qingfeng Chen, Lei Zhang, Xiaona Li

**Affiliations:** a Department of Breast Oncology, Linyi Cancer Hospital, Linyi, Shandong, China; b Department of Breast Surgery, the affiliated hospital of Qingdao University, Qingdao, China

**Keywords:** Polyphenol epigallocatechin-3-gallate, breast cancer, apoptosis, proliferation

## Abstract

The aim of the present study was to investigate the anticancer effects and potential mechanisms of polyphenol epigallocatechin-3-gallate (EGCG) on breast cancer MCF-7 cells *in vitro* and *in vivo*. Our results showed that EGCG significantly inhibited MCF-7 cell viability in a time- and dose-dependent manner. Flow cytometry analysis indicated that EGCG induced apoptosis and disrupted cell cycle progression at G2/M phase. Moreover, EGCG inhibited miR-25 expression and increased PARP, pro-caspase-3 and pro-caspase-9 at protein levels. Restoration of miR-25 inhibited EGCG-induced cell apoptosis. Furthermore, EGCG suppressed tumor growth *in vivo* by downregulating the expression of miR-25 and proteins associated with apoptosis, which was further confirmed by a reduction of Ki-67 and increase of pro-apoptotic PARP expression as determined by immunohistochemistry staining. These findings indicate that EGCG possesses chemopreventive potential in breast cancer which may serve as a promising anticancer agent for clinical applications.

## Introduction

Breast cancer is the most common malignant disease and the leading cause of cancer-related deaths among women all over the world [1]. Advances in surgery, radiotherapy, hormonal therapy and chemotherapy have improved the treatment of breast cancer, and yet more than 410,000 women still die from this disease every year [2]. The major reason for patient death is due to metastasis and resistance to current therapies that include chemotherapy, hormonal therapy and radiation. Thus, the development of novel-targeted therapeutic strategies is urgently needed to enhance the efficacy of current therapies and prolong patient survival.

(−)-Epigallocatechin-3-gallate (EGCG) is the most abundant and the most biologically active catechin in green tea and its role in cancer treatment has been intensively studied [3]. Anti-tumorigenic activities attributed to exposure to EGCG include inhibition of cell proliferation and tumor growth [4,5], induction of apoptosis and cell cycle arrest [5,6], inhibition of invasion and metastasis [7], and suppression of angiogenesis [8]. Epidemiological studies have suggested that green tea consumption is linked to a decrease in the incidence and recurrence of breast cancer [9]. Additionally, treatment with EGCG (50 mg/kg/day, 14 days) reduced the growth of MCF-7 implanted breast tumors in athymic nude mice by 40% [10], and it has been reported that catechin inhibited the proliferation of human breast cancer cells in vitro [11]. However, the underlying mechanisms are still not entirely clear.

Previous study has reported that EGCG upregulates the expression of microRNA (i.e. miR-210) by binding HIF-1α, resulting in reduced cell proliferation and anchorage-independent growth [12]. EGCG could also be an effective natural compound targeting colorectal CSCs through suppression of Wnt/β-catenin pathway [13]. In hepatocellular carcinoma (HCC) cells, EGCG modulated the oligomeric structure of phosphofructokinase (PFK), potentially leading to metabolic stress associated apoptosis and suggesting that EGCG acts by directly suppressing PFK activity [14]. EGCG induced nuclear accumulation and transcriptional activity of nuclear factor erythroid 2-related factor 2 (NRF2), as well as binding of NRF2 to the antioxidant response element sequence located at the target gene promoters in human MCF10A breast epithelial cells. Therefore, the signal pathway that EGCG plays is different.

miR-25 is one of the miR-106b-25 cluster (miR-93, miR-25 and miR-106b) [15]. Evidence has highlighted the key regulatory roles of miR-25 in tumor progression [16]. The vast majority of studies investigating the role of miR-25 in the development of breast cancer have found that miR-25 was overexpressed in human breast cancer tissues and was elevated in the serum of patients [17,18]. A BRCA1/2A preclinical mechanistic study reported that down-regulation of miR-25 by isoliquiritigenin resulted in increased autophagic cell death by overexpression of **Unc-51-like autophagy-activating kinase (ULK)** and induced chemosensitization in MCF7/ADR breast cancer cells [19]. Another study comprehensively analyzed tumor tissue miRNA expression and patient survival collecting data from the Cancer Genome Atlas (**CGA**) which contains miRNA sequencing and overall survival datasets of 759 breast cancer patients [20]. Surprisingly, this study found that increased expression of miR-25 predicted improved breast cancer survival [20]. Michael et al. have reported that EGCG reduced the expression of miR-25, miR-92, miR-141, and miR-200a in multiple myeloma [21]. In this study, we investigated the functional mechanism of EGCG in breast cancer growth. We clarified that EGCG inhibits growth in breast cancer through inactivating miR-25.

### Materials and methods

#### Cells and cell culture

The MCF-7 cells were propagated *in vitro* in RPMI-1640 medium supplemented with 10% fetal bovine serum (FBS) and 100 U/mL penicillin (Thermo Scientific), and 100 μg/mL streptomycin (Thermo Scientific). Cells were maintained at 37°C in a humidified atmosphere containing 5% CO_2_.

#### Overexpression and inhibition of miR-25 in MCF-7 cells

Pre-miR-25 miRNA precursor molecules and miR-25 inhibitors (anti-miR-25) were purchased from Ambion (Applied Biosystems, CA, US) and were used to enforce or to antagonize mir-25 expression, respectively, at a final concentration of 100 nM. Pre-miR precursor negative control and anti-miR miRNA inhibitor negative control were obtained from Ambion (Applied Biosystem, CA, US). 1 × 10^6^ cells were transfected using Neon® Transfection System (Invitrogen, CA, US), (1 pulse at 1050 V, 30 ms), and the transfection efficiency evaluated by flow cytometric analysis relative to a **FAM (5-Carboxyfluorescein) dye** labeled anti-miR negative control reached 85–90%.

#### Quantitative real-time PCR

For quantitation of individual miR-25, cDNA was synthesized from total RNA using miRNA-specific primers according to the manufacturer’s protocol for the TaqMan® MicroRNA assay (Applied Biosystems). Briefly, reverse transcriptase reactions were performed in 15 μL containing 5 μL of purified total RNA, 50 nM stem-loop RT primers, 1× RT buffer, 0.25 mM each of dNTPs, 3.33 U/μL Multiscribe™ Reverse Transcriptase, and 0.25 U/μL of RNase inhibitor. The reverse transcription reaction mixtures were incubated for 30 min at 16°C, 30 min at 42°C, 5 min at 85°C, and then cooled. RT products were further diluted four times with RNase-free water. Real-time PCR was performed using TaqMan® Universal PCR Master Mix. A 20-μL PCR reaction included 1 μL of diluted RT product, 1× of the corresponding miRNA assay primers, and 1× TaqMan®Universal PCR Master Mix (Applied Biosystems). Real-time PCR reactions were performed using the Applied Biosystems 7900HT (Applied Biosystems) with the following conditions: 95°C for 10 min, followed by 50 cycles of 95°C for 15 s, and 60°C for 1 min. All reactions were run in duplicate. Real-time PCR was performed using the Applied Biosystems 7900HT (Applied Biosystems). Data were analyzed with SDS software, version 2.3 (Applied Biosystems), to determine C_t_ by the second derivative max method. Relative quantities of miRNAs were calculated using the ΔΔ*C*_t_ method with reference U6 snRNA (average C_*t*_ values of all four independent U6 snRNA probes) as internal controls. The ΔC_t_ (C_*t*_ target miRNA – C_*t*_U6 snRNA average) relative expression = 2^−ΔΔCt^, where ΔΔCt = (ΔCt of the sample) – (ΔCt of the calibrator).

#### Western blot analysis

Cells were lysed and sonicated in RIPA lysis buffer (Cell Signaling, Danvers, MA) containing phosphatase inhibitor cocktail (Roche Diagnostics, Indianapolis, IN). Lysates were centrifuged at 10,000 × g for 10 min at 4°C. Pellets were discarded and the total protein concentration of the supernatants was determined by DC Protein Assay Reagents Package kit (Bio-Rad, Hercules, CA). The samples were boiled for 5 min at 97°C. Twenty-five micrograms of protein was loaded per lane for SDS-PAGE gel separation and then transferred to nitrocellulose membrane. Membranes were blocked with Blocking Buffer (Thermo Scientific, Waltham, MA) and then incubated overnight at 4°C with primary antibodies against caspase-3,9 and poly (ADP-ribose) polymerase-1 (PARP-1). Following the incubation of membranes with secondary antibodies conjugated to horseradish peroxidase (Cell Signaling) for 1 h at room temperature. Labeled proteins were detected with chemiluminescence detection reagent (Cell Signaling). The membrane was transferred to x-ray film and exposed. The intensity of the protein bands in films was quantified using ImageJ software and the data were reported as % of control.

#### Cell proliferation assays

A cell proliferation assay measuring total DNA content was performed using crystal violet dye. Briefly, MCF-7 cells (1 × 10^5^) were plated in 12-well plates and allowed to adhere overnight. Cells were treated with EGCG (0.5–20 μg/ml) in serum-free media containing 0.5% bovine serum albumin (BSA) and, after 72 h, the media was removed and wells for each condition, in triplicate, were fixed and stained. A staining solution containing 20% methanol and 0.5% crystal violet was added to each well, incubated for 30 min at room temperature, and rinsed thoroughly. After air-drying, a constant volume of 15% acetic acid solution was added to each well to solubilize the dye. The plates were placed on an orbital shaker until all dye was completely dissolved. Equal aliquots from each well were transferred to a 96-well plate and their absorbance was read at 570 nm. To study the effect of miR-25 sliencing on cell proliferation, when the cells reached 70–80% confluence, they were transfected with miR-25 inhibitor (100nM) or negative control and incubated for 72 h. To study the effect of miR-25 on EGCG-induced cell grow inhibition, MCF were transfected with pre-miR-25 (100nM) or negative control for 24 h before the treatment of EGCG.

#### Assessment of cell cycle progression and apoptosis by flow cytometry

To assess cell cycle progression, MCF-7 cells were plated in triplicate wells of 24-well plates and treated with varying concentrations of EGCG (0.5–20 μg/ml) in serum-free media containing 0.5% BSA for 72 h, then the cells were collected, washed in ice-cold PBS, fixed with 70% cold ethanol and stored at 4°C. Twenty-four hours later, cells were washed with ice-cold PBS, re-suspended in PI/RNase Staining Buffer (BD Biosciences, San Jose, CA), incubated in the dark for 15 min at RT, and then analyzed by flow cytometry in a Beckman Gallios flow cytometer (Beckman Coulter Inc., Brea, CA). FCS Express software (Beckman Coulter) was used to determine the percentage of cells in each phase of the cell cycle. Apoptosis was measured with the fluorescein isothiocyanate (FITC) Annexin-V apoptosis detection kit (BD Biosciences), which uses Annexin-V and propidium iodide (PI) as the apoptotic and necrotic markers, respectively according to the manufacturer’s instructions. Apoptotic cells were analyzed in a Beckman Gallios flow cytometer. Gallios 1.2 software (Beckman Coulter) was used to determine the percentage of apoptotic and necrotic cells.

To study the effect of miR-25 sliencing on cell apoptosis, when the cells reached 70–80% confluence, they were transfected with miR-25 inhibitor (100nM) or negative control and incubated for 72 h. To study the effect of miR-25 on EGCG-induced cell apoptosis, MCF were transfected with pre-miR-25 (100nM) or negative control for 24 h before the treatment of EGCG.

#### *Animals and* in vivo *model of MCF-7 cells*

Female CB-17 severe combined immunodeficient (SCID) mice (6 to 8-weeks old) were housed and monitored in our Animal Research Facility. All experimental procedures and protocols had been approved by the Institutional Ethical Committee (Shandong University) and conducted according to protocols approved by the National Directorate of Veterinary Services (China). Administration of EGCG to the animals began 10 days after tumor inoculation to allow the time for establishment of tumors. The mice received 100 mg/kg of EGCG dissolved in 100 μL water every 2 days by oral gavage. The mice of mock-treated group received only water. Mice were examined weekly and tumor volumes were estimated from their length (*l*) and width (*w*), as measured by calipers, using the formula, tumor volume = *l w*^2^ × 0.52. Mice were sacrificed when the tumor volume of mock group reached approximately 1000 mm^3^.

#### Tissue preparation and immunohistochemistry

Tumors from mice were excised and fixed in 10% formalin, embedded in paraffin wax blocks and sectioned. Subsequently, sections of the tumor blocks were stained with Ki67, or PARP antibody (1:500 dilution, Santa Cruz Biotechnology, Santa Cruz, CA, USA) by the pathology core facility. TUNEL analysis was performed using the *in situ* Cell Death Detection Kit (Roche, Indianapolis, IN, USA) as per the manufacturer’s protocol, and 10 randomly selected microscopic fields in each group were used to calculate the relative ratio of TUNEL-positive cells.

#### Statistical analysis

Statistical analysis was performed using the Student's t-test, and a p-value of <0.05 was considered significant. Data are expressed as the mean ± standard error of the mean (SEM). The mean value was obtained from at least three independent experiments.

## Results

### EGCG inhibits breast cancer cell growth

**Figure 1.**
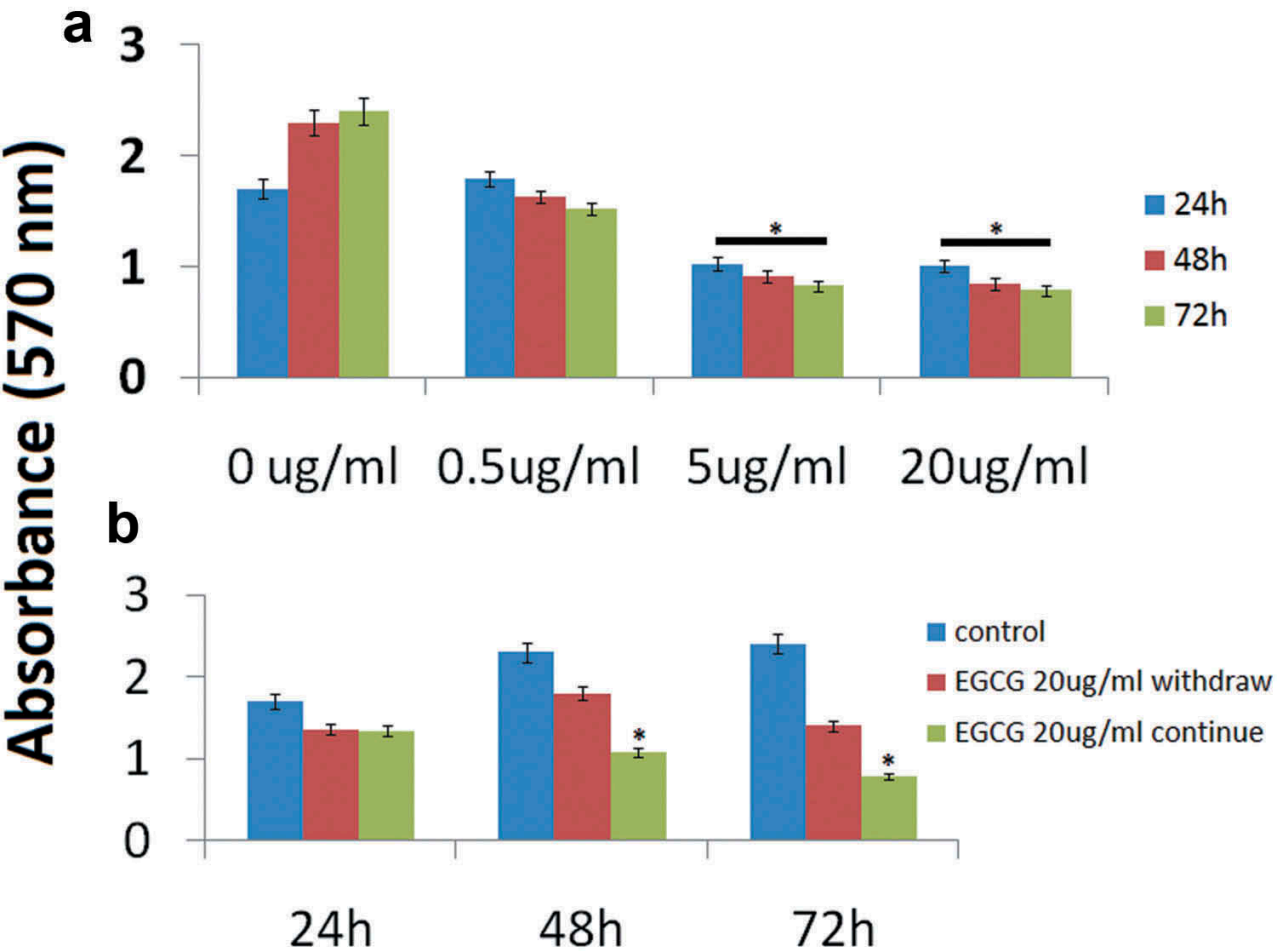
Antiproliferative effect of EGCG on MCF-7 cells *(a).* MCF-7 cells were treated with 0.5,5 and 20 μg/ml EGCG over a 24–72 h time course. Crystal violet assays were performed as described in the Methods section (* p < 0.01 vs. control at each time point). (b) After 24 h of exposure of MCF-7 cells to EGCG (20 μg/ml), the medium was replaced with fresh serum-free medium without EGCG. The assay was continued for 72 h to compare the effects of EGCG withdrawal with those of continuous EGCG exposure. (*P < 0.05 vs. EGCG withdrawn).

MCF-7 cells were plated in triplicate wells of 24-well plates and treated with varying concentrations of EGCG (0.5–20 μg/ml) in serum-free media containing 0.5% BSA. Cell quantity by crystal violet DNA staining was assessed at 24–72 h. Cell growth was inhibited by 40–75% after 72 h by 5 and 20 doses of EGCG; the antiproliferative effect of EGCG (5 and 20 μg/ml) was significant compared to the vehicle treatment at 24–72 h (Figure 1(a)). 0.5 μg/ml EGCG has no significant effect on cell viability in MCF-7 cells, so we used 5 and 20 μg/ml for further study below. In addition, 48 and 72 h has the same effect of EGCG on cell viability, we used 72 h for some experiments.

We further questioned whether or not the growth arrest induced by EGCG would persist despite withdrawal of the drug. We treated MCF-7 cells with EGCG (20 μg/ml) for 24 h and subsequently half of the EGCG-treated wells were changed to serum-free (control) media for the remainder of the experiment. The growth inhibition effect persisted even after withdrawal of EGCG, as demonstrated by decreasing crystal violet absorbance at 72 h (Figure 1(b)). However, the cells with continued EGCG treatment demonstrated significantly less cell viability than the treatment withdrawal group at 72 h.

### EGCG induces cell cycle arrest at G2/M phase

**Figure 2.**
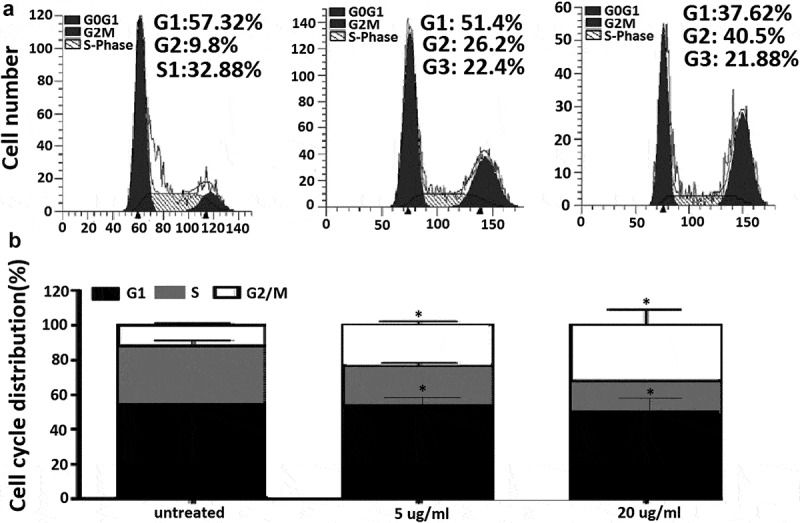
EGCG induces cell cycle arrest at G2/M phase in MCF-7 cells. (a) MCF-7 cells were treated with EGCG at 0, 5 and 20 μg/ml for 48 h. Cell cycle was analyzed by flow cytometry. (b) Quantified histograms demonstrated the effect of EGCG on MCF-7 cell cycle distribution.*p < 0.05; as compared with control group.

In order to investigate the anticancer mechanism of EGCG in MCF-7 cells, we determined the effects of EGCG on cell cycle distribution by flow cytometry analysis. As shown in Figure 2(a and b), the ratio of MCF-7 cells in G0/G1 phase slightly decreased upon EGCG treatments at non-significant level, while the ratio of cell population in S phase significantly decreased (*P*< 0.05). Compared to control cells, EGCG significantly increased percentage of G2/M phase from 11.00 ± 1.03 to 20.4 ± 2.07% and to 28.6 ± 7.46% upon 5 and 20 ug/ml of treatments, respectively (*P*< 0.05, Figure 2(b)).

### EGCG induces MCF-7 cell apoptosis

**Figure 3.**
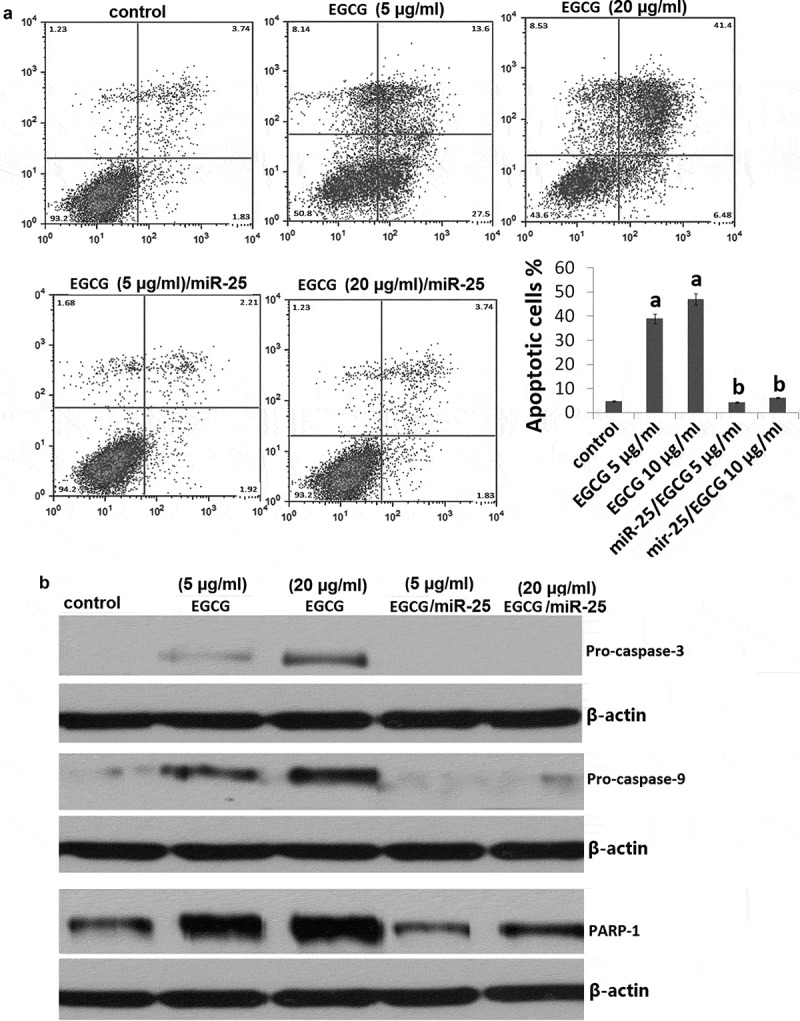
EGCG induces miR-25-dependent MCF-7 cell apoptosis. MCF-7 cells were treated with EGCG at 5 and 20 μg/ml for 72 h or transfected with mimic miR-25 before treatment with 5 and 20 μg/ml EGCG for 72 h. (a) Cell apoptosis was detected by Annexin V/PI assay; (b) Cellular proliferation was detected by crystal violet DNA staining.

We next investigated whether the effects induced by EGCG were due to apoptosis. Treatment with 5 and 20 μg/ml EGCG induced up to 24% and 43% (^a^*P*<0.05) apoptosis compared with cells treated with fresh serum-free medium without EGCG (Figure 3(a)). Western blot analysis showed that EGCG induced activation of the apoptotic-related molecules caspase-3, caspase-9 and PARP-1 72 h EGCG treatment (Figure 3(b)).

### EGCG downregulates the expression of miR-25 in breast cancer cells

**Figure 4.**
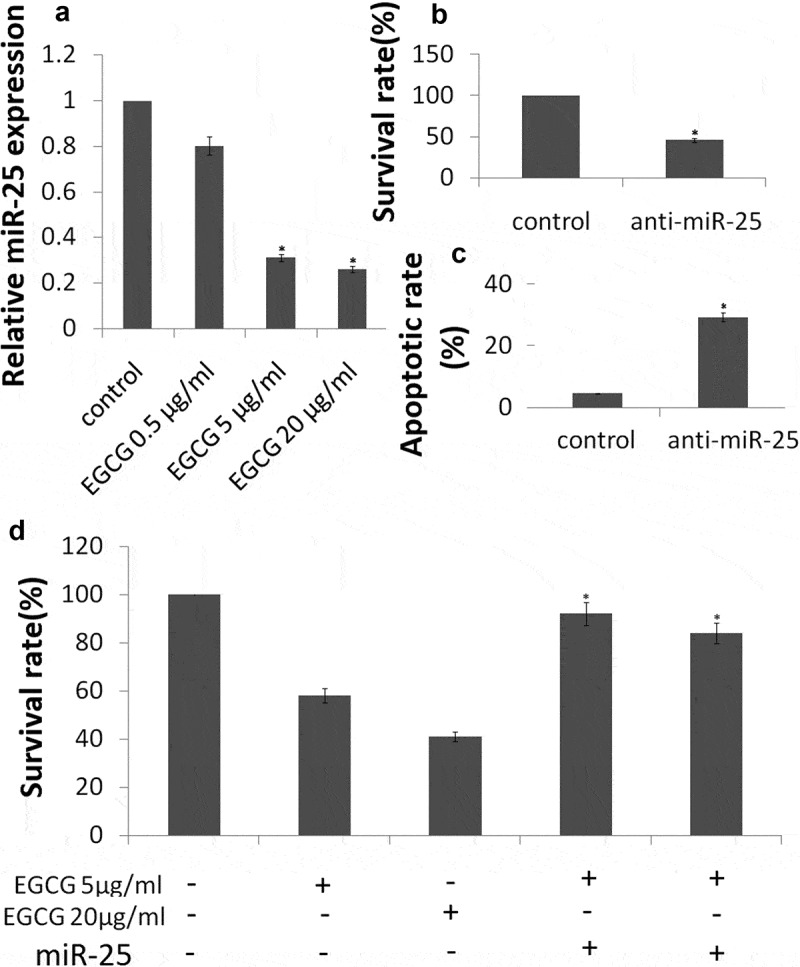
EGCG inhibits miR-25-dependent MCF-7 cell survival. MCF-7 cells were treated with EGCG at 5 and 20 μg/ml for 72 h. (a) Expression of miR-25 was detected by qRT-PCR assay; (b) Cell apoptosis was detected by Annexin V/PI assay; (c) Cellular proliferation was detected by crystal violet DNA staining. (d) MCF-7 cells were transfected with mimic miR-25 before treatment with 5 and 20 μg/ml EGCG for 72 h. Cellular proliferation was detected by crystal violet DNA staining.*P < 0.05.

To demonstrate that EGCG downregulates the expression of miR-25, an EGCG dose–response curve was generated. MCF-7 cells were treated with varying concentrations of EGCG (0.5, 5 and 20 μg/ml) for 72 h before measuring miR-25 expression with qRT-PCR (Figure 4(a)). Treatment of MCF-7 cells with EGCG at concentrations of 5, 20 μg/ml caused downregulation of the expression of miR-25 (*P*< 0.05). 0.5 μg/ml of EGCG has no significant affect on miR-25 expression.

### EGCG induces MCF-7 cell proliferation and apoptosis via downregulation of miR-25

Next, we investigated the consequences of silencing of miR-25 expression on cellular proliferation and apoptosis. Silencing of miR-25 resulted in a decrease in cellular proliferation by approximately 54.3% in MCF-7 cells (Figure 4(b),*P* < 0.05) and increase in cell apoptosis by 29.4% (Figure 4(c), *P* < 0.05). The results above showed that knockdown of miR-25 can mimic the effects of EGCG. Then, we explored whether EGCG mediate the effects via miR-25 downregulation. We transfected mimic miR-25 into the MCF-7 cells, then the cells were treated with 5 and 20μg/ml EGCG for 72 h. We found that mimic miR-25 transfection could antagonize the effects of EGCG by crystal violet DNA staining (Figure 4(d)) and Annexin V/PI assay (Figure 3(a)). In addition, mimic miR-25 inhibited EGCG-induced apoptotic-related molecules expression (Figure 3(b))

### EGCG inhibits MCF-7 xenografts in vivo

**Figure 5.**
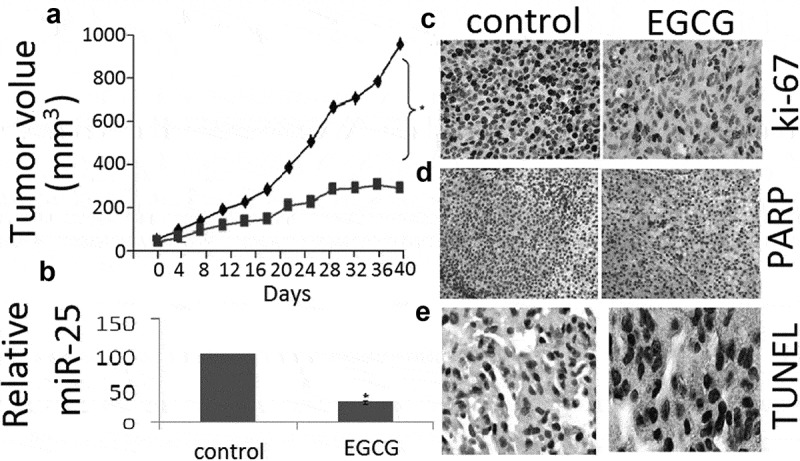
EGCG inhibits tumor growth in MCF-7 xenograft nude mice. (a) Growth curve of tumor volume in nude mice. (b) Expression ofmiR-25 in xenograft tumors as determined by qRT-PCR. (c) Expression of ki-67 in xenograft tumors as determined by IHC. (d, c) Expression of cleaved PARP in xenograft tumors as determined by IHC. (d) TUNEL analysis of xenografts after *in vivo* EGCG treatment. Data are presented as mean ± SE of three independent experiments, *p < 0.05, as compared with control group.

To evaluate the antitumor effects of EGCG *in vivo*, we examined the effects of low dose and high dose of 100 mg/kg of EGCG by oral gavage on tumor growth in a mouse tumor xenograft model. Figure 5(a) shows representative photograph of the average size of the tumor volume from each group, which indicates reduced tumor volume in EGCG-treated groups. qRT-PCR showed that the miR-25 expression was decreased by treatment with 100 µM EGCG (Figure 5(b)). IHC staining with Ki67 showed that the in vivo proliferation of MCF-7 cells was decreased by treatment with 100 µM EGCG (Figure 5(c)). Furthermore, 100 µM EGCG increased the expression level of cleaved PARP (Figure 5(d)). TUNEL staining showed that TUNEL-positive cells were increased in the EGCG-treated tumor (Figure 5(e)). In sum, these results demonstrate that EGCG could reduce the growth as well as induce apoptosis of human MCF-7 xenograft tumors.

## Discussion

In this study, we demonstrated that EGCG-induced human MCF-7 cell apoptosis and inhibited cell proliferation *in vitro* and *in vivo*. We further clarified the mechanism responsible for such cell killing. EGCG inhibited miR-25 expression in MCF-7 cells. In addition, EGCG decreased the proliferation and induced cell cycle arrest at G2/M phase and apoptosis of MCF-7 cells, which was accompanied by the activation of caspase signals. And restoration of miR-25 in MCF-7 cells reduced the EGCG-induced cell death, which was accompanied by the inactivation of caspase signals. Our findings demonstrated that EGCG might inhibit MCF-7 cells growth through miR-25 signaling pathways.

There are many reports about the effects of EGCG on cancer cell growth [24–26]. Wei et al. have reported that in breast cancer cells in vivo, EGCG exerts an anti-tumor effect through the inhibition of key enzymes that participate in the glycolytic pathway and the suppression of glucose metabolism [27]. Huang et al. have reported that EGCG suppressed the proliferation of human MCF-7 breast cancer cells and promoted apoptosis via the P53/Bcl-2 signaling pathway [28]. Luo et al. have reported that EGCG might exert influence on breast cancer progression through inhibiting focal adhesion kinase (FAK) signaling pathway [29]. However, its mechanism of action in breast cancer remains unclear.

MicroRNA is a class of non-coding, short RNA, which are responsible for regulating the expression of post-transcriptional gene. Several studies have revealed that EGCG had the potential to regulate the expression of microRNA in melanoma [30], colorectal cancer [31], cervical carcinoma [32] and lung cancer [33]. Indeed, there are some upregulated miRNAs in breast cancer, including miR-25 [34,35]. Hence, targeting the microRNAs using tea catechins may be a promising project in breast cancer therapy.

EGCG has been shown to induce apoptosis in certain tumor cells, although its precise cellular target(s) remain unclear. In our current study, we show that EGCG has anti-proliferative and pro-apoptotic effects in MCF-7 cells, inhibiting cell growth by 40–75% and inducing cell apoptosis by 24–43% in a dose-dependent fashion. These results are similar to findings in other cancer cells, including the cervical carcinoma cells [32] and lung cancer cells [33]. We additionally demonstrated EGCG treatment results in cell cycle arrest at either the G1/S or G2/M checkpoint.

miR-25 is commonly discovered in various cancers and its upregulation will promote proliferation, invasion, and migration in tumors. In breast cancer, miR-25 promoted TNBC cell proliferation in vitro and tumor growth in xenograft model, while suppression of miR-25 induced cell apoptosis [34]. Wang et al. have reported that miR-25 inhibition led to autophagic cell death by directly increasing ULK1 expression, and miR-25 overexpression was demonstrated to block ISL-induced autophagy and chemosensitization [36]. Here, we also found miR-25 was overexpressed in MCF-7 cells, and miR-25 sliencing resulted in inhibition of proliferation and induction of apoptosis in MCF-7 cells. The results above showed that knockdown of miR-25 can mimic the effects of EGCG. Our results also showed that overexpression of miR-25 significantly inhibited EGCG-induced MCF-7 cell apoptosis *in vitro*. It suggests that the mechanisms underlying EGCG suppressing the proliferation and inducing apoptosis of MCF-7 cells mainly through downregulation of miR-25.

An important finding of this study is that a oral dose of EGCG treatment at 100 mg/kg/day in drinking water significantly slows a growth curve of breast cancer in C57BL/6 female mice compared to the control group, which is characterized by 72% reduction in the tumor cross-sectional area. Clearly, oral EGCG treatment is very effective in suppressing the progression of breast cancer in a wild type immunocompetent mouse model, the mechanism of which was by inhibiting in vivo miR-25 expression, inducing cell apoptosis and inhibiting cell proliferation.

## Conclusion

EGCG has abilities in the anti-proliferation and pro-apoptosis of breast cancer cells *in vitro* and *in vivo*. Our results present evidence on the effects of EGCG against MCF-7 via modulation of miR-25 pathway. Our observation holds promise for further studies to examine the efficacy of EGCG and develop EGCG as a potential anti-cancer supplement against breast cancer.
